# Application of enhanced recovery after surgery for oral dryness prevention after endoscopic sinus surgery

**DOI:** 10.1016/j.bjorl.2024.101473

**Published:** 2024-07-20

**Authors:** Yanhong Wei, Fanglian Shu, Ying Dai, Aji Tan, Yanju Wang, Li Zhang, Yan Shu

**Affiliations:** a The People's Hospital of Suzhou New District, Department of Ophthalmology and Otorhinolaryngology, Suzhou, Jiangsu, China; b The People’s Hospital of Suzhou New District, Department of Gynecology and Obstetrics, Suzhou, Jiangsu, China; c The People's Hospital of Suzhou New District, Department of Nursing, Suzhou, Jiangsu, China

**Keywords:** Chronic rhinosinusitis, Nasal endoscopic surgery, Xerostomia, Enhanced recovery after surgery, Cluster nursing

## Abstract

**Objective:**

To investigate the efficacy of cluster nursing intervention based on Enhanced Recovery After Surgery (ERAS) for xerostomia in chronic rhinosinusitis after nasal endoscopic surgery.

**Methods:**

A total of 80 patients with chronic rhinosinusitis who underwent functional nasal endoscopic surgery between January 2020 and December 2021 were selected and randomly divided into a control group (n = 40) and an experimental group (n = 40). Patients in the control group were treated with general nursing, while ERAS-based cluster nursing intervention was adopted for the experimental group, in addition to general nursing. Xerostomia stage and comfort level were observed at 2 h, 6 h, 24 h and 48 h after surgery; negative emotions before and after nursing were also observed.

**Results:**

After the intervention, the xerostomia stage and comfort level at 6, 24 and 48 after surgery were higher in the experimental group (*p* < 0.05). Negative emotions in the experimental group were lower after nursing (*p* < 0.001). The self-rating depression scale and self-rating anxiety scale scores increased after nursing in both two groups (*p* < 0.05).

**Conclusion:**

Enhanced recovery after surgery-based cluster nursing intervention can alleviate xerostomia, improve patients' comfort levels, reduce their negative emotions and accelerate postoperative recovery.

**Level of evidence:**

2.

## Highlights

• ERAS-based cluster nursing interventions can effectively improve dry mouth.
• ERAS-based cluster nursing interventions are able to increase comfort.
• ERAS-based cluster nursing interventions can effectively reduce negative emotions.

## Introduction

Chronic rhinosinusitis, a common nasal disease in the otorhinolaryngology department, has clinical symptoms that include prolonged nasal obstruction, chronic headache, anosmia and purulent nasal discharge, which significantly affect patients' quality of life. Currently, the clinical symptoms of patients can be minimised by nasal endoscopic surgery, restoring their nasal functions.^1^, ^2^ However, due to the particularity of the anatomical position, patients' nasal cavities need to be filled with special materials after surgery for haemostasis by compression, causing patients to experience unpleasant symptoms, such as nasal obstruction, xerostomia, tears, headache, mouth breathing and swallowing disorders after surgery.^3^ Among these, xerostomia is the main symptom,^4^ which is characterised by a dry mouth, thirst, halitosis, cracked lips, dysphagia, sore throat and other manifestations.

Xerostomia may cause discomfort and distress for patients and may also affect their oral health and nutrition. Moreover, xerostomia may be associated with an increased risk of oral infections, dental caries and oral mucositis.^5^, ^6^ Therefore, it is important to prevent and alleviate xerostomia in patients after endoscopic sinus surgery.

Routine nursing measures are limited and only focus on the smooth implementation of endoscopic sinus surgery; they rarely involve attention to complications after endoscopic sinus surgery, reducing the satisfaction of patients with sinusitis. Enhanced Recovery After Surgery (ERAS) is a new method to better prevent a variety of postoperative complications and achieve early postoperative recovery by optimising perioperative treatment and reducing physical and mental post-traumatic stress in patients. Perioperative care is integral in ERAS.^7^ Routine care is mostly based on surgical procedures for intervention, and lower priority is given to the influence of individual patient factors. Compared with routine care, ERAS is better able to give comprehensive preoperative adaptive training and postoperative pacification based on individual patient factors.^8^ Cluster nursing is derived from evidence-based nursing, which integrates a series of known, effective and correlated interventions into systematic nursing protocols, contributing to specific, feasible and scientific nursing interventions.^9^ The ERAS-based cluster nursing protocols can translate ERAS theoretical knowledge into actionable, evaluable and repeatable clinical practices and realise the translation and application of ERAS guidelines and consensus, thus contributing to enhanced recovery, optimal efficacy and top-quality nursing for patients.^10^

In this study, we investigated the efficacy of cluster nursing intervention based on ERAS for preventing and alleviating xerostomia in patients with chronic rhinosinusitis after nasal endoscopic surgery. We selected xerostomia as the primary outcome measure because it is a common and bothersome symptom for patients after endoscopic sinus surgery, and may affect their oral health and nutrition. We also measured other outcomes, such as comfort level and negative emotions, to capture the overall impact of the nursing intervention on patients’ well-being. We hypothesised that the ERAS-based cluster nursing intervention would be more effective than general nursing in preventing and alleviating xerostomia, improving comfort level and reducing negative emotions in patients after endoscopic sinus surgery.

## Methods

### Study design and participants

This was a case-controlled prospective trial that used a simple random sampling method. Eighty patients with chronic rhinosinusitis who underwent endoscopic sinus surgery in our department between January 2020 and December 2021 were enrolled and divided into a control group (n = 40) and an experimental group (n = 40) according to the random number sampling method. The sample size was calculated based on the primary outcome measure (xerostomia stage). We assumed that the mean xerostomia stage score at 48 h after surgery would be 6.0 ± 2.0 in the control group and 4.5 ± 2.0 in the experimental group, based on our clinical experience and a preliminary pilot study. Using a two-sided alpha error of 0.05, a power of 80%, and an allocation ratio of 1:1, we calculated that a sample size of 37 patients per group would be required to detect this difference. Considering a potential dropout rate of 10%, we decided to enroll 40 patients per group. The inclusion criteria were set as follows: (1) Patients aged 18-years or older who met the diagnostic criteria for chronic rhinosinusitis; (2) Patients with bilateral sinus lesions who had received nasal endoscopic surgery; (3) Patients who were conscious and could cooperate with oral nursing; and (4) Patients who agreed to participate in this study and signed the informed consent. The exclusion criteria involved (1) Patients with mental illness; (2) Patients with oral and metabolic diseases; and (3) Patients with xerostomia caused by other diseases. This study was conducted in accordance with the Declaration of Helsinki and approved by the ethics committee of our hospital (nº 2023-137).

### Intervention

**Fig. 1:**
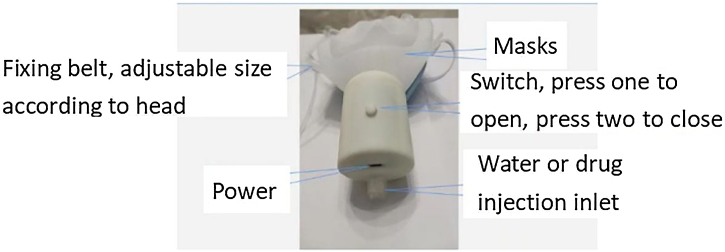
Self-made portable anti-oral dryness humidification device.

Patients in the control group were treated with general nursing, while ERAS-based cluster nursing intervention was adopted in the experimental group in addition to general nursing. The intervention included preoperative assessment and training, perioperative diet instruction, postoperative oral nursing, the application of a self-made portable anti-xerostomia device (Fig. 1), the use of appropriate traditional Chinese medicine techniques, postoperative fluid management, and psychological nursing. The details of the intervention are described in Table 1.^11^, ^12^, ^13^, ^14^ In this study, all patients received standard postoperative pain management with ibuprofen extended-release tablets.

**Table 1:** ERAS-based cluster nursing intervention.

Intervention	Content	Time
Preoperative assessment and training	Assess the patients’ oral health, hydration status, and psychological state. Instruct the patients to perform breathing and swallowing training in case of nasal obstruction.^11^	Two days before surgery, three times a day, two hours after meals.
Perioperative diet instruction	According to the Clinical Guidelines for Enhanced Recovery After Surgery (ERAS) in China (2021)^12^ Instruct the patients to abstain from eating six hours before surgery and from drinking two hours before surgery, but allow them to drink 250 mL of 10% glucose solution two hours before surgery. Instruct the patients to drink warm water two hours after surgery when their vital signs are stable, and their swallowing function is normal.	Before and after surgery.
Postoperative oral nursing	Assess the patients’ xerostomia stage two hours after surgery. Instruct the patients to gargle with warm water, brush their teeth, and apply moisturizing agents to their oral cavity.	Two hours after surgery, and then every morning and evening, and before and after meals.
Application of self-made portable anti-xerostomia device	Apply a mask with a humidifying device and a temperature-regulating device to the patients’ face (see Fig. 1 for device details). Use cold boiled water as the humidification solution. Adjust the pressure and temperature of the air according to the patients’ comfort.	Two hours after surgery, and then as needed.
Application of appropriate traditional Chinese medicine techniques	Massage the acupoints of Shuiquan and Yuji gently and then heavily for five minutes each time.^13^ Perform this when the patients have soreness, swelling pain and mild pain at the acupoints or lower extremities.	Within 48 h after surgery, once an hour when the patients are awake.
Postoperative fluid management	Assess the patients’ xerostomia stage and hydration status. Instruct the patients to drink at least 50 mL of water per hour if their xerostomia stage is 1–2, and at least 70 mL of water per hour if their xerostomia stage is 3‒4. Perform intravenous infusion according to the patients’ condition, and avoid excessive fluid infusion. An intravenous infusion was performed according to a patient’s condition, and fluid infusion was offered based on the basal amount of 1–2 mL (kg/h) within three days after surgery.^14^	Within 48 h after surgery.
Psychological nursing	Provide health education, music therapy, and emotional support to the patients. Help the patients to reduce their anxiety, depression, and stress. Enhance their confidence and cooperation in the recovery process.	Throughout the perioperative period.

### Outcome measures

The primary outcome measure was the xerostomia stage, which was assessed by the linear visual analogue scale of subjective xerostomia.^15^ The scale includes 8 items, namely mouth, throat, lip, dry tongue, degree of dysphagia, diminished taste, narrative difficulty and drinking water at night. Each of these items adopts a 5-level scoring system. A score of 0-points means no impact at all, while a score of 4-points indicates a significant impact. The higher the score, the more severe the patients’ xerostomia. Evaluations were performed at 2 h, 6 h, 24 h and 48 h after surgery.

The secondary outcome measures were the patient’s comfort level and negative emotions. The comfort level was evaluated by Kolcaba’s General Comfort Questionnaire,^16^ which includes physiological, psychological, environmental and sociocultural dimensions. The questionnaire includes 26 items, each of which is scored using a Likert scale ranging from 1 to 4 points. For inverse problems, ‘1’ indicates ‘strongly agree’, while ‘4’ reflects ‘strongly disagree’. The higher the score, the higher the comfort level of patients. The scale has high reliability and validity, with a CVI of 0.86 and Cronbach's α of 0.92.^17^ Evaluations were performed at 6, 24 and 48 after surgery. Negative emotions were assessed by the Self-rating Depression Scale (SDS) and Self-rating Anxiety Scale (SAS), which are widely used tools for measuring depression and anxiety levels, respectively. The higher the score, the more negative the emotions. Evaluations were performed before and after nursing.

### Statistical analysis

The SPSS 21.0(IBM, Armonk, NY, USA) software package was used for data analysis. Counting data were expressed as a frequency (%). Chi-Square analysis was used for categorical variables, and Fisher's exact test (Monte Carlo simulation) was used when the expected frequency was less than 5. The Mann–Whitney *U* test was used to analyse the differences between groups and to rank the data. Measurement data were expressed as mean ± standard deviation, differences between groups were compared using repeated measures analysis of variance and *p* < 0.05 indicated statistically significant differences.

## Results

### Baseline characteristics of study participants

The control group consisted of 26 men and 14 women aged (36.18 ± 13.42) years, while the experimental group included 31 men and 9 women aged (39.85 ± 14.26) years. The SDS before nursing in the control and experimental groups was 28.33 ± 6.89 vs. 29.43 ± 7.29 (*p* = 0.49), indicating no difference in the baseline sinus condition between the two groups.

There were no statistical differences in the general data between the two groups, including gender, age, comorbidities (anxiety disorders, depression, smoking status, diabetes, aspirin allergy) and asthma and allergic rhinitis complications (*p* > 0.05) (Table 2). In this study, haemostatic sponges were used as nasal tamponade in both patient groups. Ibuprofen extended-release tablets were used to relieve postoperative pain. The specific surgical procedures were tailored to the individual patient's disease severity and anatomy; there were no significant differences between the two groups in the proportion of patients undergoing more extensive procedures such as frontal sinus drill-out.

**Table 2:** Comparison of general data.

Characteristics	Control group (n = 40)	Experimental group (n = 40)	*p*-value
Gender, n (%)			0.112
Male	26 (65.0)	31 (77.5)	
Female	14 (35.0)	9 (22.5)	
Age, years, mean ± SD	36.18 ± 13.42	39.85 ± 14.26	0.239
Comorbidities, n (%)			
Anxiety disorders	3 (7.5)	2 (5.0)	1.000
Depression	2 (5.0)	1 (2.5)	1.000
Smoking	8 (20.0)	10 (25.0)	0.592
Diabetes	4 (10.0)	3 (7.5)	1.000
Aspirin allergy	1 (2.5)	2 (5.0)	1.000
Asthma, n (%)	5 (12.5)	6 (15.0)	0.745
Allergic rhinitis, n (%)	12 (30.0)	14 (35.0)	0.626
SDS score, mean ± SD	28.33 ± 6.89	29.43 ± 7.29	0.490

SD, Standard Deviation; SDS, Self-rating Depression Scale.

### Comparison of postoperative xerostomia and comfort

The comparison showed no statistically significant difference in the xerostomia stage between the two groups 2 h after surgery (*F* = 3.652, *p* = 0.060). There was a statistically significant difference in the xerostomia stage between the two groups at 6 h, 24 h and 48 h after surgery (*p* < 0.05), and the effect in the experimental group was significantly higher than in the control group (Table 3).

**Table 3:** Changes in the xerostomia stage of patients in the two groups at 2 h, 6 h, 24 h, and 48 h after surgery.

Group	2 h after surgery	6 h after surgery	24 h after surgery	48 h after surgery
Experimental group	2.1 ± 0.67	2.65 ± 0.58	1.63 ± 0.49	1.08 ± 0.27
Control group	2.35 ± 0.48	3 ± 0.56	2.1 ± 0.5	1.6 ± 0.5
*F*	3.652	7.614	18.549	34.745
p	0.060	0.007	<0.001	<0.001

When compared, comfort levels showed a statistically significant difference between the two groups at 6 h, 24 h and 48 h after surgery (*p* < 0.001). It was significantly higher in the experimental than in the control group (Table 4).

**Table 4:** Changes in the comfort level of patients in the two groups at 6 h, 24 h, and 48 h after surgery.

Group	n	6 h after surgery	24 h after surgery	48 h after surgery
Experimental group	40	60 ± 6.98	81.13 ± 4.9	95.75 ± 3.78
Control group	40	50.15 ± 6.22	71.53 ± 7.81	84.58 ± 5.05
*F*		44.397	43.43	125.583
*p*		<0.001	<0.001	<0.001

### Preoperative and postoperative negative mood comparison

According to the comparison, the two groups showed no statistically significant differences regarding negative emotions on admission (SDS: [*t* = 0.694, *p* =  0.490]; SAS: [*t* = 0.694, *p* =  0.490]). After nursing, the two groups showed statistically significant differences in negative emotions (SDS: [*t* = −6.636, *p* < 0.001] and SAS: [*t* = −4.140, *p* ≤ 0.001]), and the effect in the experimental group was significantly higher than in the control group (Table 5).

**Table 5:** Changes in negative emotions of patients in the two groups after admission.

	Experimental Group	Control Group	*t*	*p*
SDS before nursing	29.43 ± 7.29	28.33 ± 6.89	0.694	0.490
SDS after nursing	56.58 ± 3.95	63.3 ± 5.04	−6.636	< 0.001
*t*	−18.217	−31.638		
p	<0.001	<0.001		
SAS before nursing	29.43 ± 7.29	28.33 ± 6.89	0.694	0.490
SAS after nursing	57.98 ± 4.11	62.38 ± 5.32	−4.140	<0.001
*t*	−21.217	−44.749		
p	<0.001	<0.001		

## Discussion

### Enhanced recovery after surgery-based cluster nursing intervention can alleviate postoperative xerostomia and improve postoperative comfort level

Based on ERAS, the duration of perioperative fasting for patients was shortened, and patients were asked to drink 10% glucose (250 mL) 2 h before surgery and to drink water 2 h after surgery, once their vital signs were stable. These actions can help patients to avoid becoming dehydrated to some extent, enhance the reserve of carbohydrates in their bodies, improve their anti-stress capabilities and alleviate their xerostomia. Early postoperative assessment of the xerostomia stage and early attention to oral nursing can relieve not only patients' symptoms of xerostomia but also a sore throat resulting from general anaesthesia with intubation, thereby helping to prevent postoperative infection. Preoperative breathing and swallowing training for patients with nasal obstruction can lower the amount of water taken away along the respiratory tract due to mouth breathing, maintain normal swallowing function after surgery, and help patients take in food and water, further helping them to rehydrate and alleviating postoperative xerostomia. According to Table 2, through the implementation of appropriate nursing measures, the xerostomia of patients in the experimental group was significantly less severe than for patients in the control group at 6 h, 24 h and 48 h after surgery. This suggests that ERAS-based cluster nursing intervention had an obvious effect on alleviating xerostomia in patients after nasal endoscopic surgery. The results of the study showed that the postoperative comfort level of patients in the experimental group, who received the series of ERAS-based cluster nursing measures aimed at alleviating postoperative discomfort, was significantly higher compared with the control group. This suggests these nursing interventions played a positive role in improving patient comfort and aiding postoperative recovery in this setting. The results of the study showed that the postoperative comfort level of patients in the experimental group was significantly higher compared with the control group.

### Enhanced recovery after surgery-based cluster nursing intervention can reduce postoperative negative emotions in patients

Psychological intervention was performed for patients throughout the perioperative period, including a full explanation of knowledge related to the disease, interpretation of the precautions related to surgery to help patients fully understand the surgical and anaesthesia methods, as well as early notification of the nursing measures that would be implemented to address postoperative discomfort to improve patients’ postoperative psychological tolerance. Standardised music therapy was offered for patients to complete the postoperative recovery in a pleasant environment. The results of this study showed that the negative emotions of patients in the experimental group were significantly lower than in the control group. The psychological intervention delivered by nursing staff for patients throughout the process facilitated nurse-patient communication and helped patients develop a positive state of mind, thus reducing their postoperative negative emotions,^18^ which is conducive to their postoperative recovery.

### Limitations

This study has some limitations. First, the sample size was small (n = 80), and there may have been issues with the representativeness of the sample. While we included patients of both genders across a wide age range, our single-center study may not fully capture the diversity of the chronic rhinosinusitis patient population in terms of factors such as socioeconomic status, and disease severity. Our sample size calculation was based on the primary outcome measure (xerostomia stage) and existing literature on the expected effect size. We believe that our sample size of 80 patients (40 in each group) was sufficient to detect a clinically meaningful difference between the two groups while minimising the risk of Type I and II errors. However, we agree that a larger sample size could further increase the power and generalisability of our findings. Second, the study included a limited number of outcome indicators, primarily focusing on subjective measures of xerostomia severity, comfort level, and negative emotions. The study content could be enriched by including objective measurements to evaluate the effectiveness of our interventions. Objective scale assessment tools include the mastication-stimulated salivary flow-rate method, as well as the dynamic salivary secretion measurement method. Although these can reflect the level of salivary secretion and the degree of oral wetness, it is difficult to formulate a gold standard for objective evaluation of xerostomia because of the obvious individual variability of the threshold of xerostomia, as well as the differences in the intensity, duration, frequency and degree of distress that xerostomia can cause among patients, even under the same value. In future studies, further improvements will be made based on these considerations to improve the quality of the study.

## Conclusion

In summary, implementing ERAS-based cluster nursing intervention protocols can effectively relieve postoperative xerostomia, improve the postoperative comfort level and accelerate the postoperative recovery of patients with chronic rhinosinusitis after nasal endoscopic surgery. Nevertheless, these nursing measures will be more convenient for clinical nurses to implement and better suited for clinical popularisation if they are formulated into nursing procedures for preventing xerostomia.

## Authors’ contributions

(I) Conception and design: Wei YH, Shu FL and Dai Y.

(II) Administrative support: Tan AJ and Zhang L.

(III) Provision of study materials or patients: Wang YJ and Shu Y.

(IV) Collection and assembly of data: Wei YH, Zhang L and Shu Y.

(V) Data analysis and interpretation: Tan AJ, Shu FL and Dai Y.

(VI) Manuscript writing: All authors.

(VII) Final approval of manuscript: All authors.

## Ethics approval and consent to participate

This study was conducted in accordance with the declaration of Helsinki.This study was conducted with approval from the Ethics Committee of The People's Hospital of Suzhou New District (2023-137). Written informed consent was obtained from all participants.

## Availability of data and materials

All data generated or analyzed during this study are included in this published article.

## Consent for publication

The manuscript is not submitted for publication or consideration elsewhere.

## Funding

We acknowledge the fund for the Medical Key Supported Discipline Project of Suzhou (SZFCXK202110); The Medical and Health Science and Technology Program of Suzhou Nen District. (2020Q009); Patent Project: National Utility Model Patent (ZL201721153978.7)

## Conflicts of interest

The authors declare no conflicts of interest.
